# Clinicians who primarily practice in nursing homes and outcomes among residents with urinary tract infection or pneumonia

**DOI:** 10.1017/ash.2023.527

**Published:** 2023-12-06

**Authors:** Melissa R. Riester, Cody M. Douglas, Joe B.B. Silva, Rupak Datta, Andrew R. Zullo

**Affiliations:** 1 Department of Epidemiology, Brown University School of Public Health, Providence, RI, USA; 2 Center for Gerontology and Healthcare Research, Department of Health Services, Policy, and Practice, Brown University School of Public Health, Providence, RI, USA; 3 Veterans Affairs Connecticut Healthcare System, West Haven, CT, USA; 4 Department of Internal Medicine, Yale School of Medicine, New Haven, CT, USA; 5 Center of Innovation in Long-Term Services and Supports, Providence Veterans Affairs Medical Center, Providence, RI, USA

## Abstract

**Objective:**

Assess the association between clinicians who primarily practice in nursing homes (NHs) and 14-day resident outcomes following initial antibiotic dispensing for pneumonia or urinary tract infection (UTI).

**Design:**

Retrospective cohort.

**Setting:**

U.S. NHs.

**Participants:**

NH residents aged ≥65 years who were prescribed antibiotics for pneumonia or UTI between 1 January 2016 and 30 November 2018.

**Methods:**

Medicare fee-for-service claims were linked to Minimum Data Set data. Clinicians who primarily practiced in NHs prescribed ≥90% of Part D dispensings to NH residents. Outcomes included death, all-cause and infection-specific hospitalization, and subsequent antibiotic dispensing. Adjusted risk ratios were estimated using inverse-probability-of-treatment-weighted (IPTW) modified Poisson regression models adjusting for 53 covariates.

**Results:**

The study population included 28,826 resident-years who were prescribed antibiotics for pneumonia and 106,354 resident-years who were prescribed antibiotics for UTI. Among the pneumonia group, clinicians who primarily practiced in NHs were associated with a greater risk of death (RR 1.3; 95%CLs 1.0, 1.6), lower risks of all-cause (RR 0.9; 95%CLs 0.8, 0.9) and infection-specific hospitalization (RR 0.8; 95%CLs 0.7, 0.9), and similar risk of subsequent antibiotic dispensing (RR 1.0; 95%CLs 1.0, 1.1) after IPTW. No meaningful associations were observed between clinicians who primarily practiced in NHs and outcomes among the UTI group.

**Conclusions:**

Clinicians who primarily practiced in NHs were associated with a lower risk of hospitalization but greater risk of mortality for NH residents with pneumonia. Further examination is needed to better understand drivers of differences in infection-related outcomes based on clinicians’ training and primary practice setting.

## Introduction

Infections are particularly challenging to evaluate and manage among older adults residing in nursing homes (NHs). Infections can present atypically among NH residents, such as blunted or absent fever with serious bacterial infections.^1–4^ In addition, residents with cognitive impairment may have difficulties with recognizing and communicating symptoms. The choice of antibiotics to treat bacterial infections is complicated by NH residents’ increased vulnerability to antibiotic-related adverse effects, such as acquisition of multidrug-resistant organisms or development of *Clostridioides difficile* infection.^2,5–12^

Clinicians who primarily practice in NHs may be particularly attuned to the unique considerations with diagnosing and treating infections among NH residents. Residents treated by these clinicians may have reduced infection-related adverse outcomes. However, clinicians who primarily practice in NHs could also be associated with challenges in care coordination (i.e., with specialists outside of the NH). Prior literature has examined relationships between clinicians who primarily practice in NHs and potentially avoidable hospitalizations, healthcare costs, clinical quality measures, and potentially harmful medication prescribing, although the impact on infection-related outcomes is understudied.^6,13–23^ If differences in antibiotic prescribing patterns and clinical outcomes differ for residents treated by clinicians who do versus do not primarily practice in NHs, then it could be beneficial for antibiotic stewardship programs to strategically adapt interventions and target them to clinicians based on clinician effort in NHs to improve infection-related outcomes.^24^

The objective of this study was to examine the association between clinicians who primarily practiced in NHs and infection-related outcomes among U.S. NH residents who were treated with antibiotics for urinary tract infection (UTI) or pneumonia. We focused on UTI and pneumonia because they represent the most common bacterial infections in NHs.^9,11,25,26^ We hypothesized that residents whose initial antibiotic treatment was prescribed by clinicians who primarily practiced in NHs would have improved infection-related outcomes compared to residents whose initial antibiotic treatment was prescribed by clinicians who did not practice primarily in NHs.

## Methods

### Study design and data sources

This observational study linked Medicare claims to minimum data set (MDS) version 3.0 clinical assessments from 2015 through 2018. Medicare data included the Medicare Beneficiary Summary File for demographic and plan enrollment information, Medicare Provider Analysis and Review claims for inpatient hospital stays, a hybrid 20% random sample of Part D claims plus all prescription drug dispensings to long-stay NH residents, and Medicare Data on Provider Practice and Specialty (MD-PPAS) for clinician-specific information. Facility-level information was ascertained from Certification and Survey Provider Enhanced Reports (CASPER). We identified long-stay NH residents using the validated Residential History File.^27^ The Brown University Institutional Review Board approved the study. The need for informed consent was waived. Additional information on the study methods is available in the Brown Digital Repository.^28^

### Study population

#### Overview

Eligible participants were long-stay (≥100 days in any U.S. NH) residents aged ≥65 years with Medicare fee-for-service. Residents were prescribed systemic antibiotics for UTI or pneumonia by a physician or advanced practice professional (nurse practitioner, physician assistant) between 1 January 2016 and 30 November 2018. Since residents could be represented once per year during the study period, analyses were conducted at the person-period (“resident-year”) level. We excluded resident-years with antibiotic dispensings from multiple prescribers on the index antibiotic dispensing date and those with zero days of follow-up time for any outcome of interest. A graphical depiction of the study design is outlined in eFigure 1.

#### Identification of antibiotics to treat infections

Since information on medication indication is not available in Part D claims, we inferred antibiotics that were used to treat UTI or pneumonia based on the dates of Part D dispensings and MDS assessments. First, we identified oral and injectable (i.e., intravenous, intramuscular) antibiotic dispensings from Medicare Part D claims during the study period. Then, we ascertained quarterly MDS assessments with documented diagnoses of UTI or pneumonia during the study period. MDS assessments with both UTI and pneumonia diagnoses were excluded. We linked antibiotic dispensings to MDS assessments if the dates of the antibiotic days of therapy (antibiotic dispensing date plus the days supplied) overlapped with the dates of the MDS assessment period (eFigure 2).^28^

After linking dispensings to MDS data, we excluded antibiotic dispensings for residents without continuous Part A enrollment or with any Medicare Advantage enrollment in the 3 months prior to the dispensing date, missing prescriber characteristics, or missing covariate information in the 6 months prior to the dispensing date. Since our aim was to focus on infections that were diagnosed and treated in the NH, we excluded antibiotic dispensings if residents had a hospitalization in the 3 days prior to the dispensing date. Finally, we randomly sampled one infection (UTI or pneumonia) per resident per year.

#### Clinicians who primarily practiced in nursing homes

We defined clinicians who primarily practiced in NHs as prescribers for whom ≥90% of all dispensings (not just antibiotics) associated with their National Provider Identifier number were for NH residents. The patient residence code in Part D claims was used to identify dispensings to NH residents. Clinician type was reassessed annually.

#### Outcomes

The outcomes of interest were all-cause mortality, all-cause hospitalization, hospitalization for septicemia or infection (which included UTI for the UTI group or pneumonia for the pneumonia group), and subsequent antibiotic dispensing in the 14 days following the index antibiotic dispensing date. Hospitalization for specific causes was ascertained based on the International Classification of Diseases, Tenth Revision, codes listed in the principal discharge diagnosis position.^28^ Since multiple prescribers typically care for NH residents (including on-call clinicians), residents were considered to have a subsequent antibiotic dispensing regardless of whether the prescription was written by the initial prescriber. Additional information on outcome ascertainment is presented in eTable 1.

#### Covariates

We ascertained information on 53 covariates (eTable 2).^28^ Resident demographics (age, sex, and race/ethnicity) were collected from the Medicare Beneficiary Summary File. Information on functional status, health instability, cognitive impairment, multimorbidity, and clinical conditions was ascertained from the MDS assessment closest to the UTI or pneumonia diagnosis, up to 185 days prior to (and inclusive of) the index antibiotic dispensing date. Resident information related to bladder and bowel devices, special treatments (e.g., oxygen therapy), and programs (e.g., isolation or quarantine) were only observed at the time of the assessment that documented the UTI or pneumonia diagnosis, as these characteristics are most clinically relevant to treatment decisions at the time of active infection. The age, sex, and type (e.g., physician in geriatric medicine, advanced practice professional) of prescribers were ascertained from the MD-PPAS for the year of the index antibiotic dispensing. CASPER data were used to identify 11 facility characteristics for the year of the index antibiotic dispensing. If facility information was not available for that year, we ascertained information for the prior year.

### Follow-up

Start of follow-up (time zero) began on the day of the first antibiotic dispensing for the UTI or pneumonia episode. Follow-up continued until an outcome event (each evaluated separately), death (for outcomes other than mortality), disenrollment from Medicare fee-for-service Parts A or D, or administrative end of follow-up (end of the study period [December 31, 2018] or 14 days after time zero), whichever occurred first.

### Statistical analysis

We summarized resident characteristics and initial antibiotic therapy by prescriber type. Antibiotics for UTI and pneumonia were classified separately into 10 categories (eTable 3). We described the number of antibiotic dispensings, days of antibiotic therapy, route of administration, and top 10 initial antibiotic regimens from the index antibiotic dispensing date.

Crude cumulative incidences, risk differences (RDs), and risk ratios (RRs) for outcomes were reported. Adjusted RRs were estimated using stabilized inverse-probability-of-treatment-weighted (IPTW) modified Poisson regression models with robust 95% confidence limits (CLs) accounting for 53 covariates.^29^ The IPTW Poisson regression models’ predicted values were used to estimate 14-day RDs. The propensity scores used to construct IPTWs were estimated using a logistic regression model, where having a clinician who primarily practiced in NHs (versus not) prescribe the initial antibiotic therapy was the outcome (eFigure 3).^30^ The IPTWs were properly distributed for the UTI (mean [SD], 1.0 [0.3]; minimum–maximum, 0.4–7.0) and pneumonia groups (mean [SD], 1.0 [0.3]; minimum-maximum, 0.5–3.9). Covariate balance was assessed using standardized mean differences.

#### Sensitivity analysis

A quantitative bias analysis was conducted by estimating E-values for crude and adjusted RRs.^31^

#### Stability analyses

To capture sequelae that may have resulted after the completion of the initial course of antibiotics, we conducted a stability analysis by ascertaining outcomes in the 30 days following the index antibiotic dispensing date. After reviewing our findings, we also conducted a post hoc stability analysis for the pneumonia group where adjusted RDs and RRs for 14-day outcomes were re-estimated using IPTW and inverse-probability-of-censoring-by-death-weighted Poisson regression models to account for the competing risk of death.

## Results

### Study population

The study population included 94,880 long-stay NH residents (106,354 resident-years) who were prescribed antibiotics for UTI and 27,481 residents (28,826 resident-years) who were prescribed antibiotics for pneumonia (eFigure 4). Clinicians who primarily practiced in NHs prescribed the initial antibiotic therapy to 26.8% and 33.1% of resident-years, respectively. Among 28,997 prescribers overall, 13.9% switched type during the study period. Prescribers wrote prescriptions for a median (quartile 1, quartile 3) of 189 (117, 290) Medicare beneficiaries annually.

Nearly all resident characteristics were well-balanced by prescriber type before IPTW (Table 1). However, for the UTI and pneumonia groups, resident-years with clinicians who primarily practiced in NHs were more likely to have female clinicians, reside in for-profit NHs, NHs in the South, NHs in urban areas, and with a greater number of beds, percent occupancy, and acuity compared to resident-years with clinicians who did not primarily practice in NHs (Table 2). All covariates were well balanced after IPTW (eTable 2).

**Table 1.** Characteristics of long-stay nursing home residents with urinary tract infection and pneumonia, by clinician type

	UTI		Pneumonia	
Characteristic^a^	Clinicians who did not primarily practice in NHs (*n* = 77,799 Resident-years)	Clinicians who primarily practiced in NHs (*n* = 28,555 Resident-years)	Clinicians who did not primarily practice in NHs (*n* = 19,282 Resident-years)	Clinicians who primarily practiced in NHs (*n* = 9,544 Resident-years)
Age, years, mean (SD)	84.2 (8.4)	83.1 (8.5)	84.3 (8.7)	83.6 (8.8)
Female sex	62,153 (79.9%)	22,229 (77.8%)	13,147 (68.2%)	6,331 (66.3%)
Race/ethnicity				
Non-Hispanic White	67,473 (86.7%)	23,424 (82.0%)	16,423 (85.2%)	7,836 (82.1%)
Non-Hispanic Black	5,499 (7.1%)	3,388 (11.9%)	1,562 (8.1%)	1,029 (10.8%)
Hispanic	3,139 (4.0%)	1,214 (4.3%)	804 (4.2%)	465 (4.9%)
Other	1,688 (2.2%)	529 (1.9%)	493 (2.6%)	214 (2.2%)
Year				
2016	33,839 (43.5%)	11,633 (40.7%)	6,655 (34.5%)	3,097 (32.4%)
2017	25,782 (33.1%)	9,351 (32.7%)	6,900 (35.8%)	3,363 (35.2%)
2018	18,178 (23.4%)	7,571 (26.5%)	5,727 (29.7%)	3,084 (32.3%)
ADL status^b^				
Independent to limited assistance	16,483 (21.2%)	5,479 (19.2%)	4,010 (20.8%)	1,947 (20.4%)
Extensive assistance	31,976 (41.1%)	11,546 (40.4%)	7,076 (36.7%)	3,408 (35.7%)
Extensive dependency	29,340 (37.7%)	11,530 (40.4%)	8,196 (42.5%)	4,189 (43.9%)
Health instability^c^				
None	38,642 (49.7%)	14,068 (49.3%)	5,753 (29.8%)	2,965 (31.1%)
Minimal	25,177 (32.4%)	9,305 (32.6%)	6,974 (36.2%)	3,374 (35.4%)
Low	11,144 (14.3%)	4,044 (14.2%)	5,025 (26.1%)	2,337 (24.5%)
Moderate to very high	2,836 (3.6%)	1,138 (4.0%)	1,530 (7.9%)	868 (9.1%)
Cognitive impairment^d^				
Cognitively intact	22,691 (29.2%)	8,234 (28.8%)	6,061 (31.4%)	2,837 (29.7%)
Mildly impaired	18,734 (24.1%)	6,733 (23.6%)	4,417 (22.9%)	2,184 (22.9%)
Moderately impaired	28,982 (37.3%)	10,679 (37.4%)	6,368 (33.0%)	3,185 (33.4%)
Severely impaired	7,392 (9.5%)	2,909 (10.2%)	2,436 (12.6%)	1,338 (14.0%)
Multimorbidity, mean (SD)^e^	2.4 (1.8)	2.4 (1.7)	2.6 (1.8)	2.6 (1.7)
**Conditions**				
ADRD	47,310 (60.8%)	17,986 (63.0%)	11,038 (57.2%)	5,724 (60.0%)
Anemia	26,162 (33.6%)	10,096 (35.4%)	7,365 (38.2%)	3,620 (37.9%)
Arthritis	7,747 (10.0%)	2,291 (8.0%)	1,562 (8.1%)	661 (6.9%)
Atrial fibrillation	9,723 (12.5%)	3,434 (12.0%)	2,716 (14.1%)	1,235 (12.9%)
Benign prostatic hyperplasia	3,514 (4.5%)	1,447 (5.1%)	1,142 (5.9%)	574 (6.0%)
Cancer	2,276 (2.9%)	841 (2.9%)	712 (3.7%)	299 (3.1%)
Chronic lung disease	18,604 (23.9%)	6,799 (23.8%)	7,873 (40.8%)	3,782 (39.6%)
Coronary artery disease	9,759 (12.5%)	3,534 (12.4%)	2,523 (13.1%)	1,212 (12.7%)
Diabetes	29,072 (37.4%)	11,004 (38.5%)	6,577 (34.1%)	3,292 (34.5%)
Heart failure	19,724 (25.4%)	7,167 (25.1%)	6,716 (34.8%)	3,150 (33.0%)
Hypertension	63,524 (81.7%)	23,204 (81.3%)	15,688 (81.4%)	7,825 (82.0%)
GERD	8,779 (11.3%)	2,829 (9.9%)	1,912 (9.9%)	856 (9.0%)
Multidrug resistant organism	1,722 (2.2%)	651 (2.3%)	354 (1.8%)	143 (1.5%)
Neurogenic bladder	5,028 (6.5%)	1,915 (6.7%)	742 (3.8%)	357 (3.7%)
Obstructive uropathy	2,568 (3.3%)	1,129 (4.0%)	379 (2.0%)	201 (2.1%)
Renal insufficiency/ESRD	6,121 (7.9%)	2,341 (8.2%)	1,737 (9.0%)	821 (8.6%)
Respiratory failure	1,685 (2.2%)	802 (2.8%)	1,526 (7.9%)	884 (9.3%)
Stroke	9,278 (11.9%)	3,663 (12.8%)	2,159 (11.2%)	1,162 (12.2%)
Venous thromboembolism	1,198 (1.5%)	518 (1.8%)	420 (2.2%)	201 (2.1%)
**Appliances**				
Indwelling catheter	9,576 (12.3%)	3,835 (13.4%)	1,187 (6.2%)	608 (6.4%)
External catheter	147 (0.2%)	75 (0.3%)	32 (0.2%)	17 (0.2%)
Ostomy	1,808 (2.3%)	696 (2.4%)	399 (2.1%)	192 (2.0%)
Intermittent catheterization	698 (0.9%)	280 (1.0%)	59 (0.3%)	35 (0.4%)
**Special treatments, procedures, and programs**				
Oxygen therapy	11,467 (14.7%)	4,118 (14.4%)	7,955 (41.3%)	3,771 (39.5%)
Quarantine or isolation precautions	746 (1.0%)	317 (1.1%)	206 (1.1%)	86 (0.9%)
Tracheostomy care	314 (0.4%)	206 (0.7%)	366 (1.9%)	291 (3.0%)
Ventilator	192 (0.2%)	132 (0.5%)	219 (1.1%)	171 (1.8%)
BiPAP/CPAP	347 (0.4%)	80 (0.3%)	93 (0.5%)	44 (0.5%)
Intravenous medications	4,548 (5.8%)	1,933 (6.8%)	1,960 (10.2%)	1,014 (10.6%)
Hospital use in past 90 days	8,740 (11.2%)	3,182 (11.1%)	2,877 (14.9%)	1,324 (13.9%)
ICU use in past 90 days	2,417 (3.1%)	917 (3.2%)	1,005 (5.2%)	509 (5.3%)

Note. ADL, activities of daily living; ADRD, Alzheimer’s disease and related dementias; BiPAP/CPAP, bilevel positive airway pressure/continuous positive airway pressure; ESRD, end-stage renal disease; GERD, gastroesophageal reflux disease; ICU, intensive care unit; NH, nursing home; SD, standard deviation; UTI; irinary tract infection. Clinicians who primarily practiced in nursing homes were defined as prescribers with ≥90% of all dispensings (not only antibiotics) to nursing home residents. Clinician type was calculated yearly. We sampled one pneumonia/UTI infection per resident per year, so residents could be represented multiple times during the study period.

a Characteristics are reported as number (%) unless otherwise noted.

b Measured using the Minimum Data Set Morris 28-point scale of Independence in Activities of Daily Living and categorized as: 0 to 14 (independent to limited assistance required), 15 to 19 (extensive assistance required), 20 or higher (extensive dependency).

c Measured using Minimum Data Set Changes in Health, End-Stage Disease, and Symptoms and Signs score, a 6-point scale of health instability categorized as: 0 (no instability), 1 (minimal health instability), 2 (low health instability), and 3 or higher (moderate to very high health instability).

d Measured using Minimum Data Set Cognitive Function Scale, a 4-point scale of cognitive function categorized as: 0 (cognitively intact), 1 (mildly impaired), 2 (moderately impaired), and 3 (severely impaired).

e Measured using the Charlson comorbidity index.

**Table 2.** Characteristics of clinicians and facilities among long-stay nursing home residents with urinary tract infection and pneumonia, by clinician type

	UTI		Pneumonia	
	Clinicians who did not primarily practice in NHs (*n* = 77,799 Resident-years)	Clinicians who primarily practiced in NHs (*n* = 28,555 Resident-years)	Clinicians who did not primarily practice in NHs (*n* = 19,282 Resident-years)	Clinicians who primarily practiced in NHs (*n* = 9,544 Resident-years)
**Clinician Characteristics**				
Age				
≤44 years	14,467 (18.60%)	5,185 (18.16%)	3,476 (18.03%)	1,852 (19.40%)
45–64 years	47,988 (61.68%)	16,955 (59.38%)	12,223 (63.39%)	5,732 (60.06%)
≥65 years	13,654 (17.55%)	5,585 (19.56%)	3,251 (16.86%)	1,705 (17.86%)
Missing	1,690 (2.17%)	830 (2.91%)	332 (1.72%)	255 (2.67%)
Sex				
Female	17,825 (22.91%)	8,167 (28.60%)	4,510 (23.39%)	2,890 (30.28%)
Male	58,284 (74.92%)	19,558 (68.49%)	14,440 (74.89%)	6,399 (67.05%)
Missing	1,690 (2.17%)	830 (2.91%)	332 (1.72%)	255 (2.67%)
Clinician type				
Physician—Family or General Practice	34,234 (44.00%)	9,403 (32.93%)	7,844 (40.68%)	2,856 (29.92%)
Physician—Geriatric Medicine	2,003 (2.57%)	2,052 (7.19%)	661 (3.43%)	733 (7.68%)
Physician—Internal Medicine	28,262 (36.33%)	11,104 (38.89%)	7,718 (40.03%)	3,887 (40.73%)
Advance Practice Practitioners	5,734 (7.37%)	2,813 (9.85%)	1,542 (8.00%)	1,084 (11.36%)
Other or Missing	7,566 (9.73%)	3,183 (11.15%)	1,517 (7.87%)	984 (10.31%)
Proportion of claims to NH residents, median (Q1, Q3)	62.78% (41.36%, 78.56%)	95.89% (93.21%, 98.09%)	68.34% (48.75%, 81.34%)	95.89% (93.25%, 98.10%)
**Facility Characteristics**				
Census Region				
Midwest	25,579 (32.88%)	5,138 (17.99%)	5,910 (30.65%)	1,733 (18.16%)
Northeast	13,195 (16.96%)	7,242 (25.36%)	4,344 (22.53%)	2,818 (29.53%)
South or Missing^a^	33,108 (42.56%)	14,064 (49.25%)	7,497 (38.88%)	4,301 (45.06%)
West	5,917 (7.61%)	2,111 (7.39%)	1,531 (7.94%)	692 (7.25%)
Urban/Rural				
Urban or Missing^a^	45,018 (57.86%)	22,463 (78.67%)	13,145 (68.17%)	7,755 (81.26%)
Rural	32,781 (42.14%)	6,092 (21.33%)	6,137 (31.83%)	1,789 (18.74%)
Facility is part of a chain				
Yes or Missing^a^	41,041 (52.75%)	15,857 (55.53%)	10,888 (56.47%)	5,557 (58.23%)
No	36,758 (47.25%)	12,698 (44.47%)	8,394 (43.53%)	3,987 (41.77%)
Facility is for-profit				
Yes or Missing^a^	50,013 (64.28%)	20,407 (71.47%)	13,547 (70.26%)	7,129 (74.70%)
No	27,786 (35.72%)	8,148 (28.53%)	5,735 (29.74%)	2,415 (25.30%)
Number of beds				
1–60	12,201 (15.68%)	1,785 (6.25%)	2,132 (11.06%)	463 (4.85%)
61–90	14,728 (18.93%)	3,381 (11.84%)	3,257 (16.89%)	1,027 (10.76%)
91–120	23,229 (29.86)	8,656 (30.31%)	5,801 (30.09%)	2,901 (30.40%)
>120 or Missing^a^	27,641 (35.53%)	14,733 (51.60%)	8,092 (41.97%)	5,153 (53.99%)
Percent occupancy				
≤0.699	13,589 (17.47%)	3,452 (12.09%)	2,953 (15.31%)	996 (10.44%)
0.700–0.799	12,364 (15.89%)	3,986 (13.96%)	2,896 (15.02%)	1,342 (14.06%)
0.800–0.899	22,740 (29.23%)	8,570 (30.01%)	5,910 (30.65%)	2,986 (31.29%)
≥0.900 or Missing^a^	29,106 (37.41%)	12,547 (43.94%)	7,523 (39.02%)	4,220 (44.22%)
Acuity index^b^				
0.000–11.999 or Missing^a^	31,010 (39.86%)	8,960 (31.38%)	6,670 (34.59%)	2,909 (30.48%)
12.000–12.999	30,393 (39.07%)	12,337 (43.20%)	8,133 (42.18%)	4,067 (42.61%)
≥13.000	16,396 (21.07%)	7,258 (25.42%)	4,479 (23.23%)	2,568 (26.91%)
% residents on antibiotics				
0.00–5.99% or Missing^a^	24,643 (31.68%)	9,973 (34.93%)	6,053 (31.39%)	3,056 (32.02%)
6.00–11.99%	32,774 (42.13%)	11,990 (41.99%)	8,171 (42.38%)	4,268 (44.72%)
≥12.00%	20,382 (26.20%)	6,592 (23.09%)	5,058 (26.23%)	2,220 (23.26%)
% residents with advance directives				
0.00–29.99% or Missing^a^	22,004 (28.28%)	8,413 (29.46%)	5,332 (27.65%)	2,750 (28.81%)
30.00–74.99%	22,824 (29.34%)	8,707 (30.49%)	6,137 (31.83%)	3,085 (32.32%)
≥75.00%	32,971 (42.38%)	11,435 (40.05%)	7,813 (40.52%)	3,709 (38.86%)
% residents receiving hospice benefit				
0.00–2.99% or Missing^a^	28,468 (36.59%)	10,296 (36.06%)	7,147 (37.07%)	3,593 (37.65%)
3.00–7.49%	27,707 (35.61%)	10,548 (36.94%)	7,009 (36.35%)	3,561 (37.31%)
≥7.5%	21,624 (27.79%)	7,711 (27.00%)	5,126 (26.58%)	2,390 (25.04%)
Facility has dialysis unit or ventilator unit				
Yes	1,436 (1.85%)	814 (2.85%)	609 (3.16%)	443 (4.64%)
No or Missing^a^	76,363 (98.15%)	27,741 (97.15%)	18,673 (96.84%)	9,101 (95.36%)

Note. NH, nursing home; UTI, urinary tract infection.

a <0.3% resident-years overall had missing values for the UTI and pneumonia cohorts.

b Acuity Index is a measure of the care needed by a nursing home’s residents. It is calculated based on the number of residents needing various levels of activities of daily living (ADL) assistance (eating, toileting, transfer, bedfast, chairbound, and ambulatory), receiving special treatment (respiratory care, suctioning, intravenous therapy, and tracheostomy), and with certain conditions (dementia, psychiatric diagnosis, serious mental illness/intellectual disability, receiving physical therapy/occupational therapy/speech therapy, and tube feedings).

### Initial antibiotic therapy

Among dispensings on the index antibiotic dispensing date, the mean (SD) days of therapy was slightly lower among residents-years who were treated by clinicians who primarily practiced in NHs in the UTI and pneumonia groups (Table 3). The top three antibiotic regimens were similar by clinician type in the UTI group including fluoroquinolones, nitrofurantoin, and sulfonamide/related agents. The top three antibiotic regimens for resident-years treated by clinicians who primarily practiced in NHs in the pneumonia group were fluoroquinolones, penicillin + beta lactamase inhibitors, and third-generation cephalosporins, while the top antibiotic regimens for resident-years treated by clinicians who did not primarily practice in NHs were fluoroquinolones, other antibiotics, and macrolides (Table 3). Additional information on the proportion of resident-years who received each antibiotic class is presented in eTables 4–5.

**Table 3.** Initial antibiotics prescribed to long-stay nursing home residents with urinary tract infection and pneumonia

	*n* (%)			
	UTI		Pneumonia	
Characteristic	Clinicians who did not primarily practice in NHs (*n* = 77,799 Resident-years)	Clinicians who primarily practiced in NHs (*n* = 28,555 Resident-years)	Clinicians who did not primarily practice in NHs (*n* = 19,282 Resident-years)	Clinicians who primarily practiced in NHs (*n* = 9,544 Resident-years)
Number of antibiotic dispensings, median (Q1, Q3)	1 (1, 1)	1 (1, 1)	1 (1, 1)	1 (1, 1)
Days of therapy, mean (SD)^a^	8.3 (6.9)	7.7 (5.4)	8.9 (7.3)	8.4 (6.3)
Route of administration^b^				
Oral	71,050 (91.3%)	25,050 (87.7%)	17,349 (90.0%)	8,309 (87.1%)
Injectable	7,533 (9.7%)	3,818 (13.4%)	2,558 (13.3%)	1,583 (16.6%)
Top 10 antibiotic regimens				
1	Fluoroquinolone 20,285 (26.1%)	Fluoroquinolone 7,017 (24.6%)	Fluoroquinolone 7,549 (39.2%)	Fluoroquinolone 3,907 (40.9%)
2	Nitrofurantoin 13,933 (17.9%)	Nitrofurantoin 4,954 (17.4%)	Other antibiotics 2,055 (10.7%)	Penicillin + β lactamase inhibitors 1,054 (11.0%)
3	Sulfonamide/ Related agent 12,022 (15.5%)	Sulfonamide/ Related agent 4,028 (14.1%)	Macrolide 1,785 (9.3%)	Third-generation cephalosporin 859 (9.0%)
4	First-generation cephalosporin 7,167 (9.2%)	Third-generation cephalosporin 2,627 (9.2%)	Penicillin + β lactamase inhibitors 1,750 (9.1%)	Other antibiotics 849 (8.9%)
5	Other antibiotics 5,636 (7.2%)	Other antibiotics 2,318 (8.1%)	Tetracycline 1,524 (7.9%)	Tetracycline 800 (8.4%)
6	Third-generation cephalosporin 5,465 (7.0%)	First-generation cephalosporin 2,248 (7.8%)	Third-generation cephalosporin 1,497 (7.8%)	Macrolide 675 (7.1%)
7	Penicillin + β lactamase inhibitors 4,107 (5.3%)	Penicillin + β lactamase inhibitors 1,960 (6.9%)	First-generation cephalosporin 646 (3.4%)	Second-generation cephalosporin 249 (2.6%)
8	Penicillin 3,338 (4.3%)	Penicillin 1,103 (3.9%)	Second-generation cephalosporin 608 (3.2%)	First-generation cephalosporin 238 (2.5%)
9	Second-generation cephalosporin 2,730 (3.5%)	Second-generation cephalosporin 926 (3.2%)	Macrolide + Third-generation cephalosporin 298 (1.6%)	Macrolide + Third-generation cephalosporin 127 (1.3%)
10	Carbapenem 1,208 (1.6%)	Carbapenem 663 (2.3%)	Penicillin 288 (1.5%)	Fluoroquinolone + Third-generation cephalosporin 105 (1.1%)

Note. NH, nursing home; Q1, quartile 1; Q3, quartile 3; UTI, urinary tract infection. Reports *n* (%), unless otherwise specified. Clinicians who primarily practiced in nursing homes were defined as prescribers with ≥90% of all dispensings (not only antibiotics) to nursing home residents. Clinician type was calculated yearly. We sampled one pneumonia/UTI infection per resident per year, so residents could be represented multiple times during the study period. Medications included in the “other antibiotics” category are listed in eTable 3.

a Calculated as the sum of antibiotic days, where one day of receiving a single antibiotic counted as an antibiotic day. If residents received multiple antibiotics on the same day, we totaled the days for each antibiotic.

b Percentages may add up to greater than 100% because residents could have received multiple antibiotics with different routes of administration on the index antibiotic dispensing date.

### Incidence of infection-related outcomes

Among resident-years with clinicians who did versus did not primarily practice in NHs in the UTI group, the crude incidence of outcomes in the 14 days after the index antibiotic dispensing date was 0.2% versus 0.2% for death, 5.5% versus 4.9% for all-cause hospitalization, 2.4% versus 2.2% for hospitalization for UTI or septicemia, and 37.9% versus 37.2% for subsequent antibiotic dispensing (Table 4). In the pneumonia group, the 14-day cumulative incidence of outcomes was 1.5% versus 1.1% for death, 7.3% versus 8.6% for all-cause hospitalization, 3.6% versus 4.2% for hospitalization for pneumonia or septicemia, and 36.2% versus 36.0% for subsequent antibiotic dispensing (Table 4).

**Table 4.** Unadjusted outcomes for long-stay nursing home residents prescribed antibiotics for urinary tract infection or pneumonia

	*n* (%)			
	UTI		Pneumonia	
14-Day Outcome	Clinicians who did not primarily practice in NHs (*n* = 77,799 Resident-years)	Clinicians who primarily practiced in NHs (*n* = 28,555 Resident-years)	Clinicians who did not primarily practice in NHs (*n* = 19,282 Resident-years)	Clinicians who primarily practiced in NHs (*n* = 9,544 Resident-years)
Death	147 (0.2%)	69 (0.2%)	218 (1.1%)	147 (1.5%)
All-Cause Hospitalization	3,840 (4.9%)	1,557 (5.5%)	1,652 (8.6%)	692 (7.3%)
Hospitalization for Infection or Septicemia^a^	1,683 (2.2%)	685 (2.4%)	814 (4.2%)	342 (3.6%)
Subsequent Antibiotic Dispensing	28,907 (37.2%)	10,825 (37.9%)	6,944 (36.0%)	3,458 (36.2%)

Note. NH, nursing home; UTI, urinary tract infection. Clinicians who primarily practiced in nursing homes were defined as prescribers with ≥90% of all dispensings (not only antibiotics) to nursing home residents. Clinician type was calculated yearly. We sampled one pneumonia/UTI infection per resident per year, so residents could be represented multiple times during the study period.

a Outcome for the UTI group included hospitalization for UTI or septicemia; outcome for the pneumonia group included hospitalization for pneumonia or septicemia.

### Association between clinicians who primarily practiced in nursing homes and outcomes

**Figure 1.**
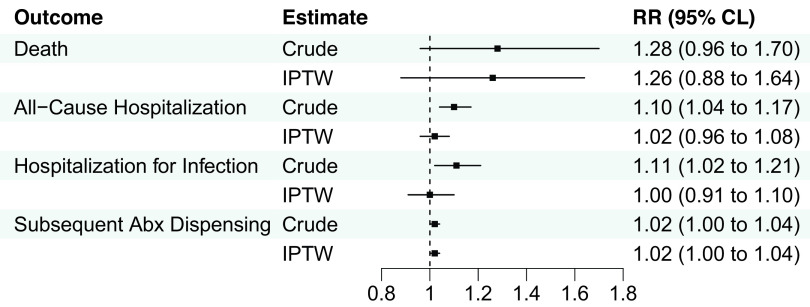
Association between clinicians who primarily practiced in nursing homes and 14-day outcomes among residents prescribed antibiotics for UTI. Abbreviations: Abx, antibiotic; CLs, confidence limits; IPTW, inverse-probability-of-treatment-weighting; RR, risk ratio; UTI, urinary tract infection. *Note*: *N* = 106,354 resident-years. Hospitalization for infection included urinary tract infection or septicemia documented in the principal discharge diagnosis position.

**Figure 2.**
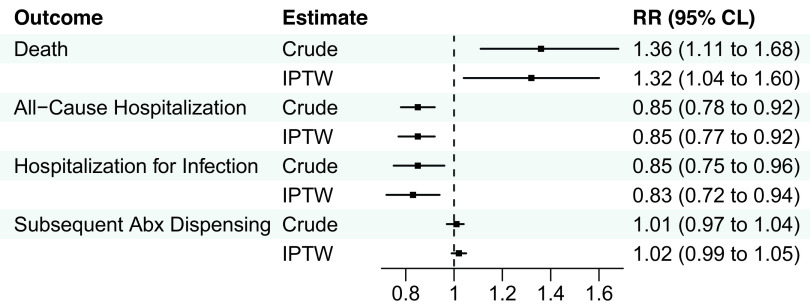
Association between clinicians who primarily practiced in nursing homes and 14-day outcomes among residents prescribed antibiotics for pneumonia. Abbreviations: Abx, antibiotic; CLs, confidence limits; IPTW, inverse-probability-of-treatment-weighting; RR, risk ratio. *Note*: *N* = 28,826 resident-years. Hospitalization for infection included pneumonia or septicemia documented in the principal discharge diagnosis position.

After IPTW, there were no significant associations between clinicians who primarily practiced in NHs and death or hospitalization outcomes in the UTI group, although there was a slightly higher risk of subsequent antibiotic dispensing (IPTW RR 1.02) among resident-years treated by clinicians who primarily practiced in NHs (Figure 1, eTable 6). Among the pneumonia group, clinicians who primarily practiced in NHs were associated with a greater risk of death (IPTW RR 1.3; 95%CLs 1.0, 1.6), lower risk of all-cause hospitalization (IPTW RR 0.9; 95%CLs 0.8, 0.9), hospitalization for pneumonia or septicemia (IPTW RR 0.8; 95%CLs 0.7, 0.9), and similar risk of subsequent antibiotic dispensing (IPTW RR 1.0; 95%CLs 1.0, 1.1) after IPTW (Figure 2, eTable 7).

### Sensitivity analysis

The E-value (lower CL) ranged from 1.6 (1.4) to 2.0 (1.2) for outcomes with significant associations after IPTW among the pneumonia group (eTable 8).

### Stability analyses

The crude 30-day cumulative incidence of outcomes is reported in eTable 9. Inferences about the association between clinician type and 30-day outcomes were not markedly different from the primary analysis for the UTI group, but the association between clinician type and mortality was no longer significant among the pneumonia group (eTables 10–11). The risk of hospitalization outcomes remained lower for residents treated for pneumonia by clinicians who primarily practiced in NHs after accounting for death as a competing risk (eTable 12).

## Discussion

In this observational study, we found that long-stay NH residents who were prescribed antibiotics for pneumonia had a higher risk of death and lower risk of hospitalization within 14 days of starting antibiotic treatment when the initial antibiotic therapy was prescribed by clinicians who primarily practiced in NHs. We found no clinically meaningful associations between clinician type and outcomes among residents who were prescribed antibiotics for UTI. This study is one of the first to examine the association between clinician effort in NH care and infection-related outcomes. However, future research is necessary to better understand why differences in outcomes existed before translating these results into practice (i.e., by tailoring antibiotic stewardship interventions to clinicians based on primary practice setting).

There is a growing body of evidence on the impact of “specialization” in NH care on resident outcomes, including potentially avoidable hospitalizations, healthcare costs, clinical quality measures, and potentially harmful medication prescribing.^13–23^ However, information related to antibiotic prescribing and infection-related outcomes is limited. Currently, no set of standardized criteria exists to define clinicians who primarily practice in NHs. A majority of other studies have defined clinicians who primarily practice in NHs based on ≥90% of professional services to NH residents using Part B claims. This study used the same threshold of ≥90%, but was based on Part D claims, which identified clinicians who prescribe most medications to long-stay NH residents. Given that bacterial UTIs and pneumonia must be treated with prescription antibiotics and our focus on prescribing, we decided that a definition using Part D dispensing claims would be most appropriate. Further examination is needed to explore and validate methods for defining clinicians who primarily practice in NHs.

In this study, NH residents who were initially treated for pneumonia by clinicians who primarily practiced in NHs had a greater risk of death and lower risk of hospitalization within 14-days, although the association between clinician type and death was not significant at 30-days. Further examination is needed to better understand the drivers of differences in outcomes. One potential contributor is the initial antibiotic regimen for pneumonia. Our results demonstrated that a greater proportion of residents treated by clinicians who primarily practiced in NHs received penicillins + beta lactamase inhibitors, third-generation cephalosporins, and a smaller proportion received macrolides. Another probable, and likely important, driver of differences in outcomes by clinician type is the presence of advanced directives, which were not captured in our data sources. Residents with do-not-hospitalize orders would have elected not to receive the higher level of care provided in the hospital. This could have contributed to the lower incidence of hospitalization and greater incidence of death if a greater proportion of residents treated by clinicians who primarily practiced in NHs documented do-not-hospitalize orders.

Future research is also needed to investigate whether the appropriateness of initial and subsequent antibiotic prescribing differs by clinician type. It may be beneficial to examine whether differences in post-prescribing modification existed by clinician type including de-escalating, shortening, or stopping antibiotic therapy. If differences exist by clinician type, antibiotic stewardship programs such as audit and feedback, education on antibiotic prescribing guidelines, and scheduled antibiotic reassessment could target prescribers separately based on clinical effort in NH care.^32,33^

### Limitations

This study has several potential limitations. These results may not generalize well to short-stay NH residents receiving post-acute care, those without Medicare fee-for-service, or with an infection other than UTI or pneumonia. Due to the nature of our data, we inferred the antibiotics that were used to treat UTI or pneumonia based on dates of antibiotic dispensings and MDS assessments, but could not confirm the indication for antibiotic therapy (i.e., using electronic health records). Additionally, we were unable to assess the appropriateness of the infection diagnosis (i.e., UTI versus asymptomatic bacteriuria), antibiotic prescribing, or severity of illness as laboratory results, microbiology data, vital signs, imaging, patient-reported symptoms (e.g., dysuria), and clinical syndromes (e.g., bronchitis, pulmonary aspiration) were unavailable. Processes related to antibiotic prescribing could not be described in further detail because they were unmeasured in the data. These include the availability of technology for clinical decision making in the NH, clinician time spent on-site in the NH, nursing staff knowledge of infections, or likelihood of nursing staff to notify prescribers of a suspected infection. E-values (lower CLs) from the quantitative bias analysis ranged from 1.6 (1.4) to 2.0 (1.2) for significant results in the pneumonia group, suggesting that results were moderately robust to residual confounding. Future work could consider using data sources with richer clinical information to incorporate covariates that were unavailable in the present study. Prescriber characteristics such as board certification, certification as a medical director, and years of experience are potentially important drivers of differences in infection-related outcomes. Finally, further validation of the definition of clinicians who primarily practice in NHs is needed to understand the impact of advanced practice professional prescribing under physicians’ National Provider Identifier numbers and the attribution of prescriptions to clinicians for medications that are administered from an emergency box or not entered electronically (as the medication may be ordered over the phone by one clinician but signed off by another clinician [e.g., Medical Director or in-house clinician]).

## Conclusion

We found significant associations between clinicians who primarily practiced in NHs (≥90% of Part D claims to NH residents) and infection-related outcomes among NH residents who were prescribed antibiotics for pneumonia. Clinicians who primarily practiced in NHs were associated with a lower risk of hospitalization but greater risk of mortality for NH residents with pneumonia. Further examination is needed to better understand drivers of differences in infection-related outcomes based on clinicians’ training and primary practice setting.

## Supporting information

Riester et al. supplementary material Riester et al. supplementary material

## Supplementary material

For supplementary material accompanying this paper visit http://doi.org/10.1017/ash.2023.527. click here to view supplementary material
