# Supplementary material for: Clinicians who primarily practice in nursing homes and outcomes among residents with urinary tract infection or pneumonia
Source: Antimicrob Steward Healthc Epidemiol. 2023 Dec 6;3(1):e253. doi: 10.1017/ash.2023.527 (PMC10762639; doi:10.1017/ash.2023.527)
Supplement: Riester et al. supplementary material [file S2732494X23005272sup001.docx]

**SUPPLEMENTARY MATERIALS**

**Title**: Clinician who primarily practice in nursing homes and outcomes among residents with urinary tract infection or pneumonia

**Authors**: Melissa R. Riester, PharmD, Cody M. Douglas, MPH, Joe B.B. Silva, ScM, Rupak Datta, MD, PhD, Andrew R. Zullo, PharmD, PhD

**eFigure 1.** Graphical depiction of the study design.

**eFigure 2.** Examples of the antibiotic dispensing selection window.

**eFigure 3.** The overlap of propensity scores.

**eFigure 4.** Flow diagram of the study population.

**eTable 1.** Description of data sources used to ascertain outcomes.

**eTable 2.** Standardized mean differences before and after inverse-probability-of-treatment-weighting.

**eTable 3.** Categorization of antibiotics into antibiotic classes.

**eTable 4.** Initial antibiotics among nursing home residents prescribed antibiotics for urinary tract infection.

**eTable 5.** Initial antibiotics among nursing home residents prescribed antibiotics for pneumonia.

**eTable 6.** Association between prescriber type and 14-day outcomes among nursing home residents prescribed antibiotics for urinary tract infection, before and after IPTW, N= 106,354 resident-years.

**eTable 7.** Association between prescriber type and 14-day outcomes among nursing home residents prescribed antibiotics for pneumonia, before and after IPTW, N= 28,826 resident-years.

**eTable 8**. E-values for 14-day outcomes.

**eTable 9.** Unadjusted 30-day outcomes for nursing home residents prescribed antibiotics for urinary tract infection or pneumonia, by prescriber type, 2016-2018.

**eTable 10.** Association between prescriber type and 30-day outcomes among nursing home residents prescribed antibiotics for urinary tract infection, before and after IPTW, N= 106,354 resident-years.

**eTable 11.** Association between prescriber type and 30-day outcomes among nursing home residents prescribed antibiotics for pneumonia, before and after IPTW, N= 28,826 resident-years.

**eTable 12.** Association between prescriber type and 14-day outcomes among nursing home residents prescribed antibiotics for pneumonia, after IPTW and IPCW due to death, N= 28,826 resident-years.

**eFigure 1. Graphical depiction of the study design.**

**
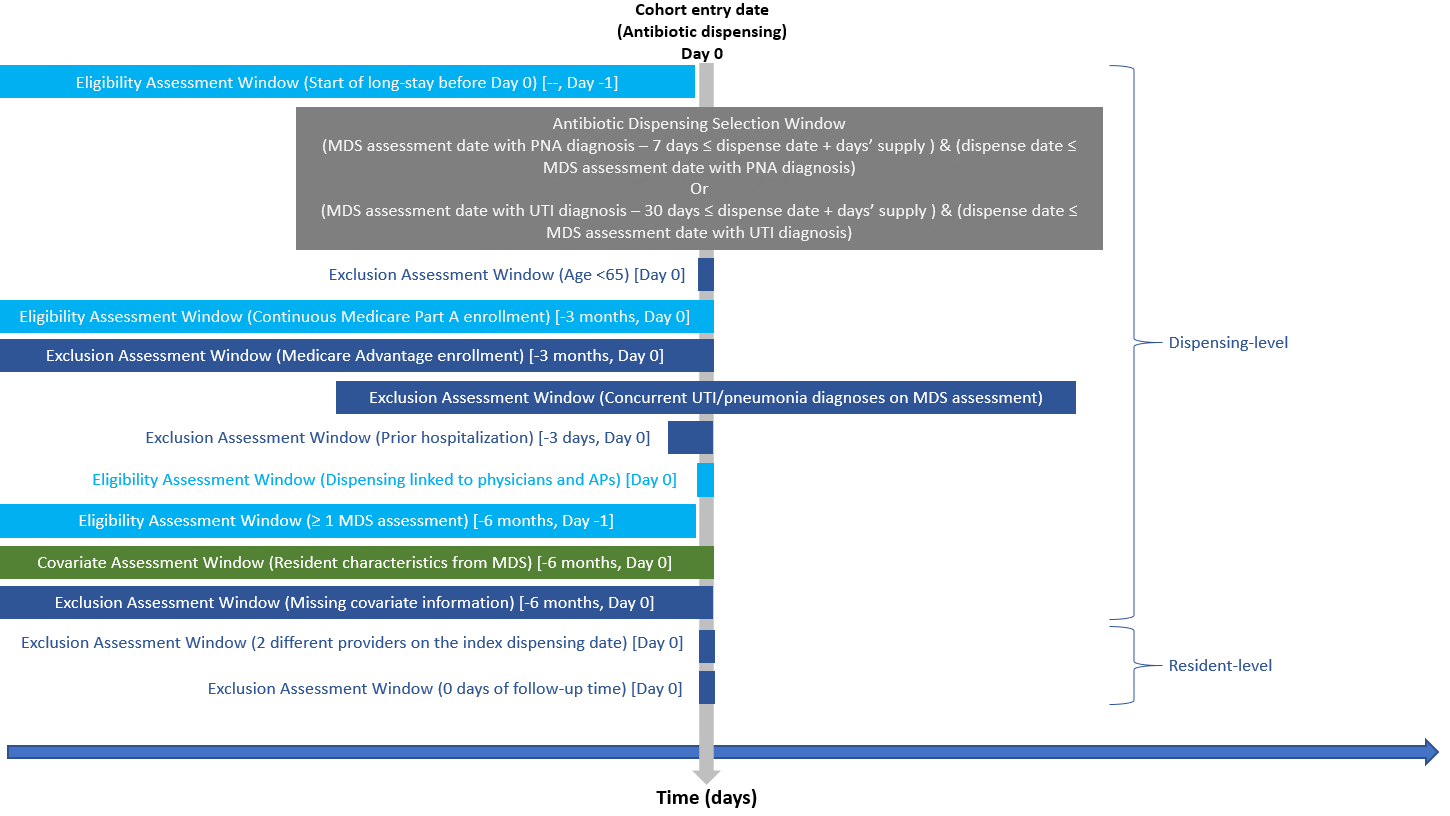
**

**Abbreviations**: AP, advanced practice professional; MDS, Minimum Data Set; PNA, pneumonia; UTI, urinary tract infection.

**Note**: Light blue bars present inclusion criteria; dark blue bars present exclusion criteria; the green bar presents the period where covariates were ascertained; the gray bar presents the criteria used to link antibiotics to infections documented on MDS assessments. Additional information on how antibiotics were linked to infections is presented in eFigure 2.

**eFigure 2. Examples of the antibiotic dispensing selection window.**


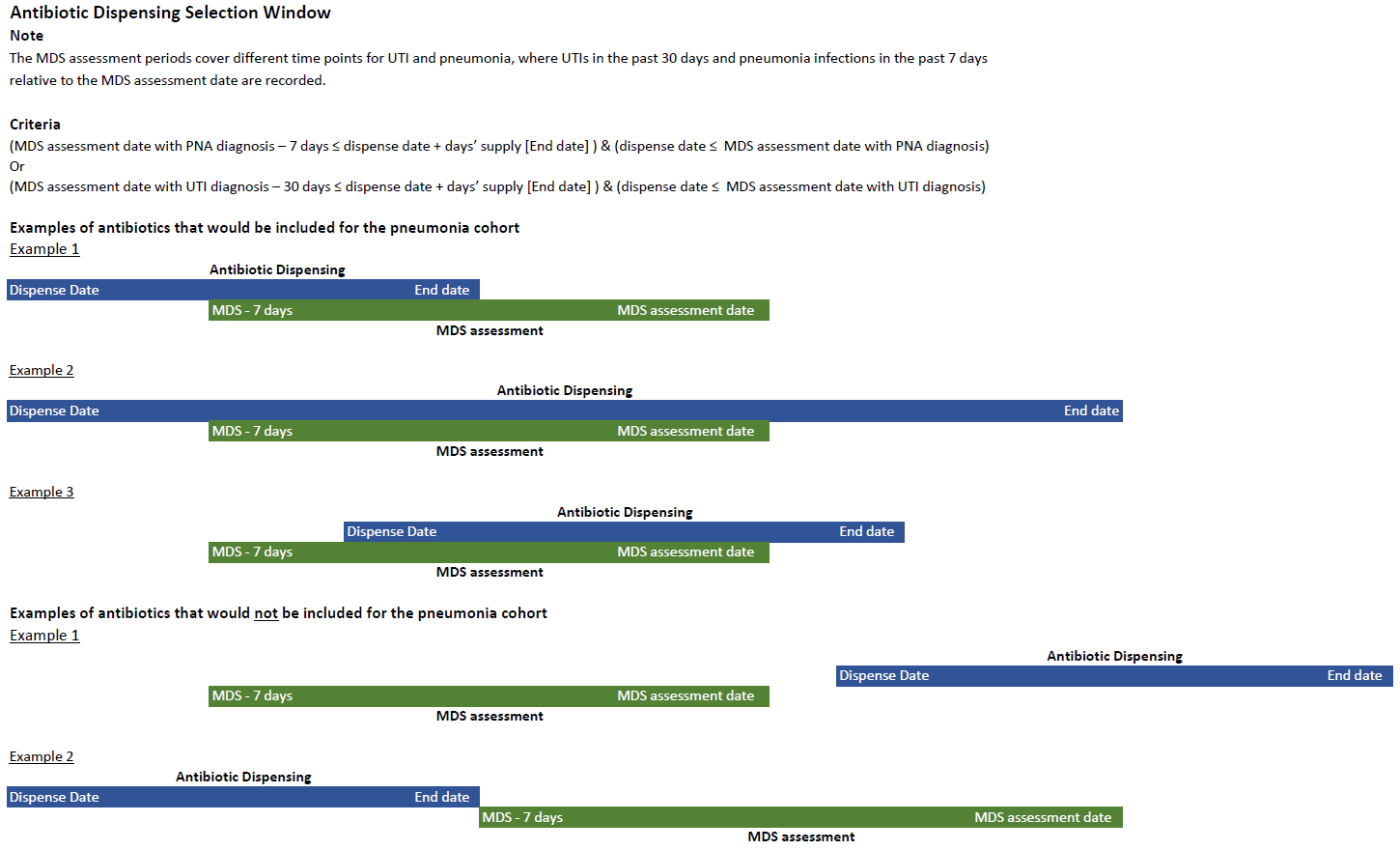


**eFigure 3. The overlap of propensity scores.**

Urinary Tract Infection

**
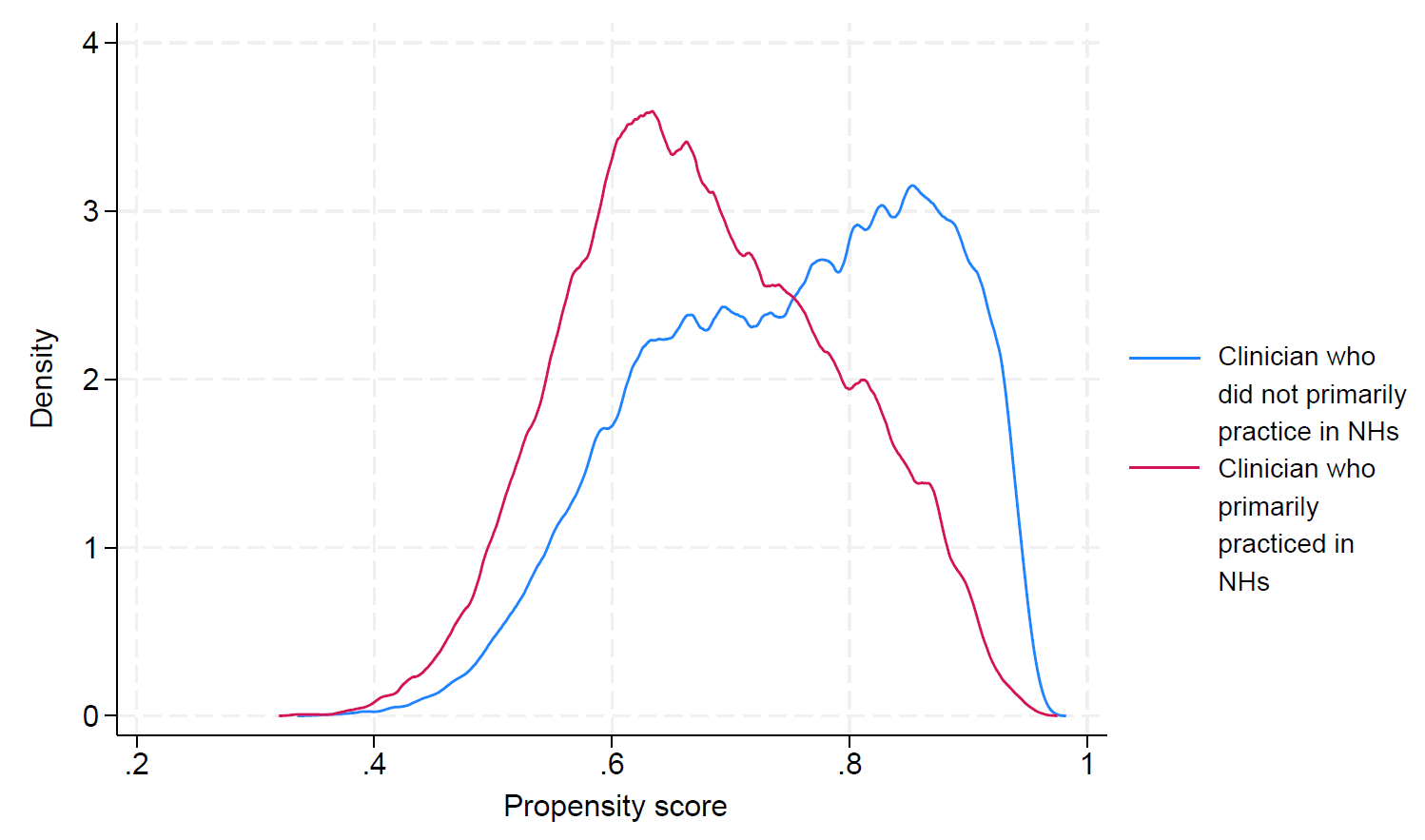
**

Pneumonia

**
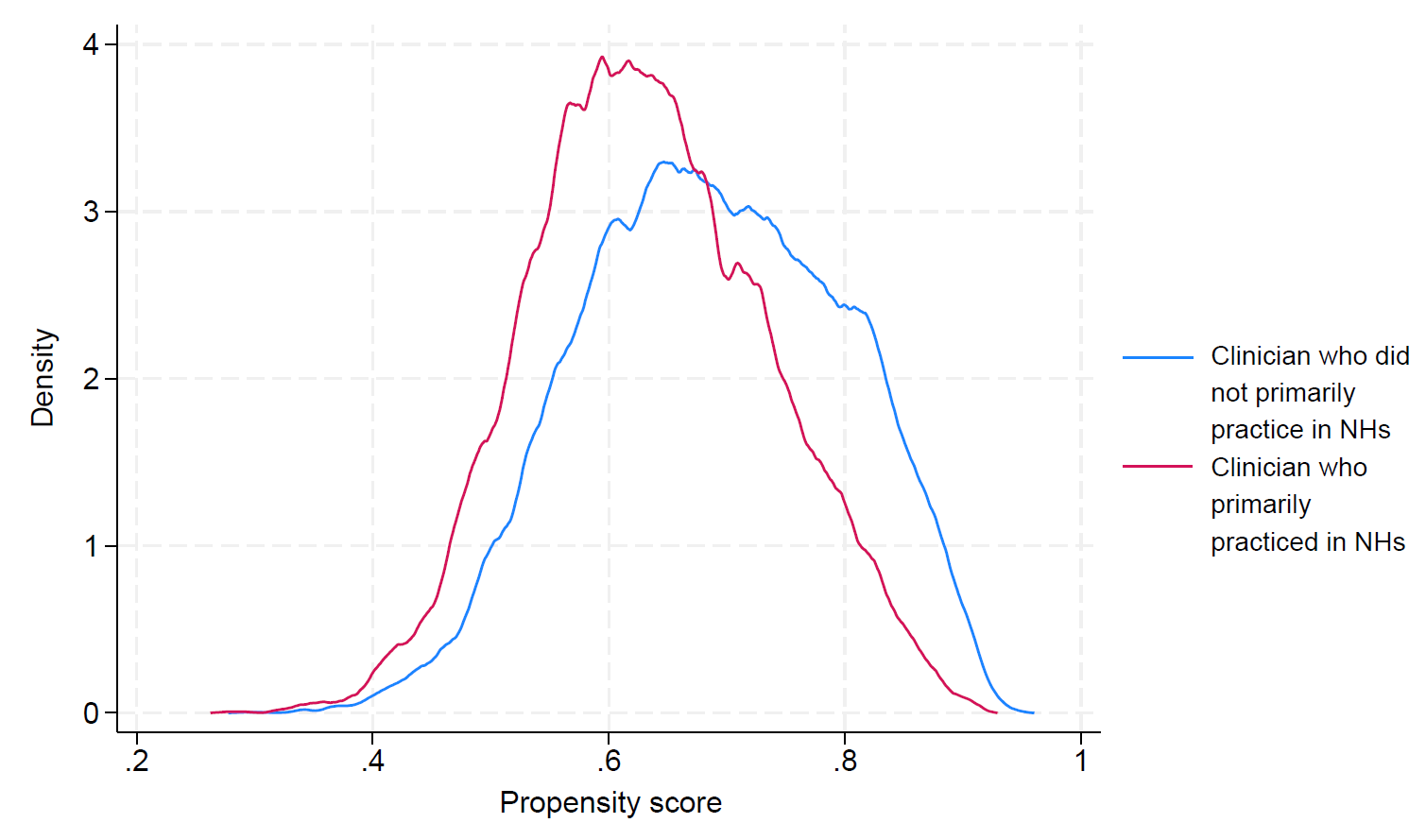
**

**Abbreviations**: NHs, nursing homes.

**eFigure 4. Flow diagram of the study population.**

**
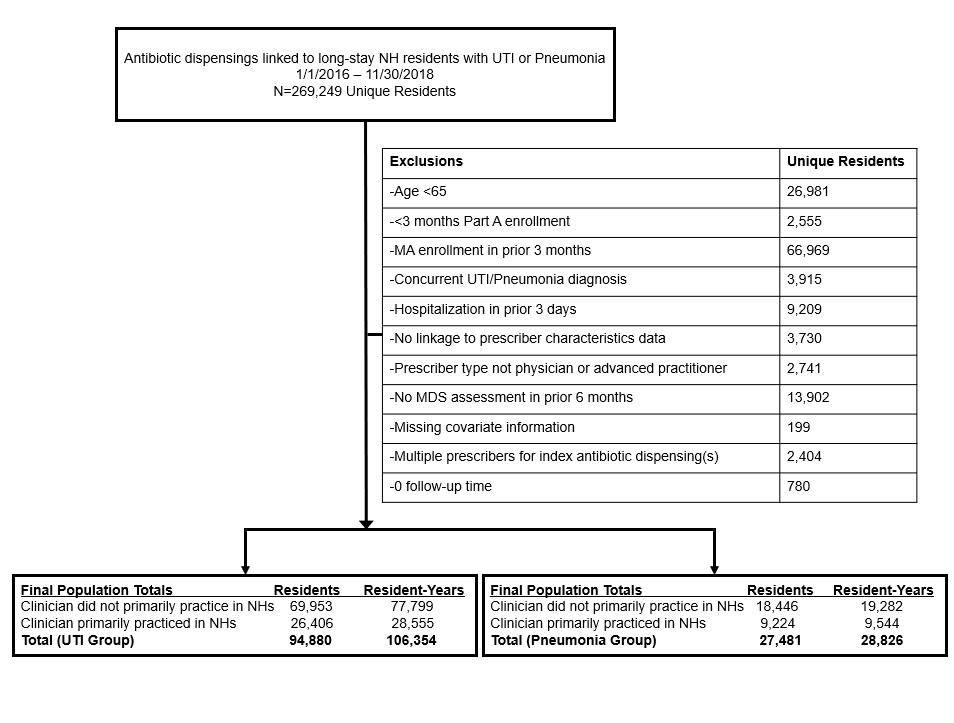
**

**Abbreviations**: MA, Medicare Advantage; MDS, Minimum Data Set; NH, nursing home; UTI, urinary tract infection.

**eTable 1. Description of data sources used to ascertain outcomes.**

| **Outcome** | **Data Source** | **Setting Where Outcomes are Ascertained** |
| --- | --- | --- |
| All-cause mortality | Medicare Beneficiary Summary File | All locations (e.g., inside the nursing home, hospital, community) |
| All-cause hospitalization | Medicare Provider Analysis and Review claims | Inpatient (short-term hospital, not long-term acute care hospital) |
| Hospitalization for septicemia or infection^a^ | Medicare Provider Analysis and Review claims | Inpatient (short-term hospital, not long-term acute care hospital) |
| Subsequent antibiotic dispensing | Part D drug dispensing claims | Nursing home or community.  Note: Drug dispensings in the emergency department, inpatient hospital, or skilled nursing facility (for post-acute care) are unobservable in Part D claims. |
| ^a^Additional information on the specific International Classification of Diseases, Tenth Revision, codes used to ascertain hospitalizations for septicemia or infection are available in the Brown Digital Repository (https://doi.org/10.26300/tfkr-f047). | | |

**eTable 2. Standardized mean differences before and after inverse-probability-of-treatment-weighting.**

|  | **UTI** | | **Pneumonia** | |
| --- | --- | --- | --- | --- |
|  | **Before IPTW** | **After IPTW** | **Before IPTW** | **After IPTW** |
| Age | -0.13 | -0.01 | -0.08 | -0.01 |
| Female sex | -0.05 | 0.00 | -0.04 | 0.00 |
| Race/ethnicity | 0.06 | 0.01 | 0.05 | 0.01 |
| Year | 0.07 | 0.00 | 0.06 | 0.00 |
| ADL status | 0.06 | 0.01 | 0.02 | 0.01 |
| Health instability | 0.01 | 0.01 | -0.01 | 0.00 |
| Cognitive impairment | 0.02 | 0.00 | 0.05 | 0.00 |
| Multimorbidity | 0.02 | 0.01 | -0.01 | 0.00 |
| **Conditions** | | | | |
| ADRD | 0.04 | 0.01 | 0.06 | 0.01 |
| Anemia | 0.04 | 0.01 | -0.01 | 0.00 |
| Arthritis | -0.07 | -0.01 | -0.04 | 0.00 |
| Atrial fibrillation | -0.01 | 0.00 | -0.03 | 0.00 |
| Benign prostatic hyperplasia | 0.03 | 0.00 | 0.00 | 0.00 |
| Cancer | 0.00 | 0.00 | -0.03 | 0.00 |
| Chronic lung disease | 0.00 | 0.01 | -0.02 | 0.01 |
| Coronary artery disease | -0.01 | 0.01 | -0.01 | 0.00 |
| Diabetes | 0.02 | 0.01 | 0.01 | 0.01 |
| Heart failure | -0.01 | 0.00 | -0.04 | 0.00 |
| Hypertension | -0.01 | 0.00 | 0.02 | 0.00 |
| GERD | -0.04 | 0.00 | -0.03 | 0.00 |
| Multidrug resistant organism | 0.00 | 0.00 | -0.03 | 0.00 |
| Neurogenic bladder | 0.01 | 0.00 | -0.01 | 0.01 |
| Obstructive uropathy | 0.03 | 0.01 | 0.01 | 0.01 |
| Renal insufficiency/ESRD | 0.01 | 0.00 | -0.01 | 0.00 |
| Respiratory failure | 0.04 | 0.00 | 0.05 | 0.00 |
| Stroke | 0.03 | 0.00 | 0.03 | 0.00 |
| Venous thromboembolism | 0.02 | 0.00 | 0.00 | 0.00 |
| **Appliances** | | | | |
| Indwelling catheter | 0.03 | 0.00 | 0.01 | 0.00 |
| External catheter | 0.02 | 0.00 | 0.00 | 0.00 |
| Ostomy | 0.01 | 0.01 | 0.00 | 0.00 |
| Intermittent catheterization | 0.01 | 0.00 | 0.01 | 0.01 |
| **Special treatments, procedures, and programs** | | | | |
| Oxygen therapy | -0.01 | 0.00 | -0.04 | 0.00 |
| Isolation or quarantine for an active infectious disease | 0.01 | 0.00 | -0.02 | 0.00 |
| Tracheostomy care | 0.04 | 0.00 | 0.07 | 0.01 |
| Ventilator | 0.04 | 0.00 | 0.05 | 0.01 |
| BiPAP/CPAP | -0.03 | 0.00 | 0.00 | 0.00 |
| Intravenous medications | 0.04 | 0.01 | 0.02 | 0.01 |
| Hospital use in past 90 days | 0.00 | 0.00 | -0.03 | -0.01 |
| ICU use in past 90 days | 0.01 | 0.01 | 0.01 | 0.00 |
| **Clinician Characteristics** | | | | |
| Clinician age | 0.04 | 0.00 | 0.08 | 0.00 |
| Clinician sex | -0.10 | 0.02 | -0.12 | 0.01 |
| Clinician type | 0.09 | 0.02 | 0.08 | 0.01 |
| **Facility Characteristics** | | | | |
| Region | 0.23 | 0.03 | 0.19 | 0.02 |
| Urban/Rural | 0.46 | 0.03 | 0.30 | 0.01 |
| Facility is part of a chain | 0.06 | 0.02 | 0.04 | 0.01 |
| Facility is for-profit | 0.15 | 0.04 | 0.10 | 0.02 |
| Number of beds | 0.42 | 0.03 | 0.33 | 0.02 |
| Percent occupancy | 0.18 | 0.02 | 0.15 | 0.01 |
| Acuity index | 0.17 | 0.05 | 0.10 | 0.02 |
| % residents on antibiotics | -0.08 | 0.00 | -0.05 | 0.01 |
| % residents with advance directives | -0.04 | -0.01 | -0.03 | -0.01 |
| % residents receiving hospice benefit | 0.00 | 0.01 | -0.03 | 0.01 |
| Facility has dialysis unit or ventilator unit | 0.07 | 0.01 | 0.08 | 0.00 |
| **Abbreviations**: ADL, Activities of Daily Living; ADRD, Alzheimer’s disease and related dementias; BiPAP/CPAP, Bilevel Positive Airway Pressure/Continuous Positive Airway Pressure; ESRD, End-Stage Renal Disease; GERD, gastroesophageal reflux disease; ICU, Intensive Care Unit; UTI; Urinary Tract Infection. | | | | |

**eTable 3. Categorization of antibiotics into antibiotic classes.**

| **Infection** | **Antibiotic Class** | **Included Antibiotics** |
| --- | --- | --- |
| UTI | Penicillins | Penicillin G, Penicillin V, Nafcillin, Oxacillin, Dicloxacillin, Ampicillin, Amoxicillin |
|  | Penicillins + β lactamase inhibitors | Ampicillin-Sulbactam, Amoxicillin-Clavulanate, Piperacillin-Tazobactam, Ticarcillin-Clavulanate |
|  | First generation cephalosporins | Cefazolin, Cephalexin, Cefadroxil |
|  | Second generation cephalosporins | Cefuroxime, Cefoxitin, Cefotetan, Cefaclor, Cefprozil |
|  | Third generation cephalosporins | Cefotaxime, Ceftriaxone, Cefpodoxime, Cefixime, Cefdinir, Cefditoren, Ceftibuten, Ceftazidime |
|  | Carbapenems | Doripenem, Ertapenem, Imipenem-cilastatin, Meropenem, Meropenem-Vaborbactam |
|  | Fluoroquinolones | Ciprofloxacin, Ofloxacin, Levofloxacin, Moxifloxacin, Gemifloxacin, Delafloxacin, Norfloxacin |
|  | Sulfonamides/Related Agents | Trimethoprim, Sulfamethoxazole-Trimethoprim, Sulfadiazine, Sulfisoxazole |
|  | Nitrofurantoin | Nitrofurantoin |
|  | Other antibiotics | Aminoglycosides: Gentamicin, Tobramycin, Amikacin, Neomycin, Streptomycin  Fourth generation cephalosporins: Cefepime  Lincosamides: Clindamycin, Lincomycin  Macrolides: Erythromycin, Azithromycin, Clarithromycin, Telithromycin  Other β lactams +/- β lactamase inhibitors: Aztreonam, Ceftazidime-Avibactam, Ceftaroline, Ceftolozane-Tazobactam  Glycopeptides and Lipoglycopeptides: Vancomycin, Telavancin, Dalbavancin, Oritavancin  Oxazolidinones: Linezolid, Tedizolid  Tetracyclines: Doxycycline, Minocycline, Tetracycline, Demeclocycline, Tigecycline  Other: Fosfomycin, Metronidazole, Quinupristin-Dalfopristin, Polymyxin B, Colistin, Colistimethate, Chloramphenicol, Daptomycin |
| Pneumonia | Penicillins | Penicillin G, Penicillin V, Nafcillin, Oxacillin, Dicloxacillin, Ampicillin, Amoxicillin |
|  | Penicillins + β lactamase inhibitors | Ampicillin-Sulbactam, Amoxicillin-Clavulanate, Piperacillin-Tazobactam, Ticarcillin-Clavulanate |
|  | First generation cephalosporins | Cefazolin, Cephalexin, Cefadroxil |
|  | Second generation cephalosporins | Cefuroxime, Cefoxitin, Cefotetan, Cefaclor, Cefprozil |
|  | Third generation cephalosporins | Cefotaxime, Ceftriaxone, Cefpodoxime, Cefixime, Cefdinir, Cefditoren, Ceftibuten, Ceftazidime |
|  | Fluoroquinolones | Ciprofloxacin, Ofloxacin, Levofloxacin, Moxifloxacin, Gemifloxacin, Delafloxacin, Norfloxacin |
|  | Macrolides | Erythromycin, Azithromycin, Clarithromycin, Telithromycin |
|  | Tetracyclines | Doxycycline, Minocycline, Tetracycline, Demeclocycline, Tigecycline |
|  | Intravenous glycopeptides | Vancomycin (Intravenous) |
|  | Other antibiotics | Aminoglycosides: Gentamicin, Tobramycin, Amikacin, Neomycin, Streptomycin  Fourth generation cephalosporins: Cefepime  Lincosamides: Clindamycin, Lincomycin  Other β lactams +/- β lactamase inhibitors: Aztreonam, Ceftazidime-Avibactam, Ceftaroline, Ceftolozane-Tazobactam, Doripenem, Ertapenem, Imipenem-cilastatin, Meropenem, Meropenem-Vaborbactam  Lipoglycopeptides: Telavancin, Dalbavancin, Oritavancin  Oxazolidinones: Linezolid, Tedizolid  Sulfonamides/Related agents: Trimethoprim, Sulfamethoxazole-Trimethoprim, Sulfadiazine, Sulfisoxazole  Other: Nitrofurantoin, Fosfomycin, Metronidazole, Quinupristin-Dalfopristin, Polymyxin B, Colistin, Colistimethate, Chloramphenicol, Daptomycin, Vancomycin (oral) |
| **Abbreviation**: UTI, urinary tract infection. | | |

**eTable 4.** **Initial antibiotics among nursing home residents prescribed antibiotics for urinary tract infection.**

|  | **n (%)** | |
| --- | --- | --- |
|  | **Clinicians who did not primarily practice in NHs (n=77,799 Resident-years)** | **Clinicians who primarily practiced in NHs (n=28,555 Resident-years)** |
| Antibiotic dispensings |  |  |
| 1 | 69250 (89.01) | 25036 (87.68) |
| 2 | 8021 (10.31) | 3262 (11.42) |
| 3+ | 528 (0.68) | 257 (0.90) |
| Penicillins | 3519 (4.52) | 1171 (4.10) |
| Penicillins + β lactamase inhibitors | 4378 (5.63) | 2083 (7.29) |
| First generation cephalosporins | 7401 (9.51) | 2325 (8.14) |
| Second generation cephalosporins | 2855 (3.67) | 975 (3.41) |
| Third generation cephalosporins | 6161 (7.92) | 2869 (10.05) |
| Carbapenems | 1274 (1.64) | 704 (2.47) |
| Fluoroquinolones | 21122 (27.15) | 7313 (25.61) |
| Sulfonamides/Related agents | 12409 (15.95) | 4169 (14.60) |
| Nitrofurantoin | 14452 (18.58) | 5119 (17.93) |
| Other antibiotics | 6177 (7.94) | 2564 (8.98) |
| Aminoglycosides | 897 (1.15) | 437 (1.53) |
| Fourth generation cephalosporins | 216 (0.28) | 135 (0.47) |
| Lincosamides | 386 (0.50) | 174 (0.61) |
| Macrolides | 1256 (1.61) | 354 (1.24) |
| Other β lactam +/- β lactamase inhibitors | 35 (0.04) | 23 (0.08) |
| Glycopeptides and lipoglycopeptides | 409 (0.53) | 222 (0.78) |
| Oxazolidinones | 168 (0.22) | 48 (0.17) |
| Tetracyclines | 2487 (3.20) | 1070 (3.75) |
| Other | 737 (0.95) | 310 (1.09) |
| **Note:** Percentages add up to >100% because resident-years could have been prescribed more than one antibiotic class. See eTable 3 for the list of antibiotics included in each class. | | |

|  |  |  |
| --- | --- | --- |
|  |  |  |
|  |  |  |
|  |  |  |

**eTable 5.** **Initial antibiotics among nursing home residents prescribed antibiotics for pneumonia.**

|  | **n (%)** | |
| --- | --- | --- |
| **Antibiotic Class** | **Clinicians who did not primarily practice in NHs (n=19,282 Resident-years)** | **Clinicians who primarily practiced in NHs (n=9,544 Resident-years)** |
| Antibiotic dispensings |  |  |
| 1 | 16389 (85.00) | 8064 (84.49) |
| 2 | 2579 (13.38) | 1305 (13.67) |
| 3+ | 314 (1.63) | 175 (1.83) |
| Penicillins | 332 (1.72) | 114 (1.19) |
| Penicillins + β lactamase inhibitors | 1990 (10.32) | 1195 (12.52) |
| First generation cephalosporins | 698 (3.62) | 261 (2.73) |
| Second generation cephalosporins | 740 (3.84) | 330 (3.46) |
| Third generation cephalosporins | 2177 (11.29) | 1221 (12.79) |
| Fluoroquinolones | 8041 (41.70) | 4163 (43.62) |
| Macrolides | 2333 (12.10) | 921 (9.65) |
| Tetracyclines | 1718 (8.91) | 909 (9.52) |
| Intravenous glycopeptides | 279 (1.45) | 158 (1.66) |
| Other antibiotics | 2371 (12.30) | 995 (10.43) |
| Aminoglycosides | 70 (0.36) | 43 (0.45) |
| Fourth generation cephalosporins | 146 (0.76) | 99 (1.04) |
| Lincosamides | 334 (1.73) | 164 (1.72) |
| Other β lactam +/- β lactamase inhibitors | 215 (1.12) | 125 (1.31) |
| Oxazolidinones | 36 (0.19) | 14 (0.15) |
| Sulfanamides/related agents | 763 (3.96) | 280 (2.93) |
| Other | 930 (4.82) | 332 (3.48) |
| **Note:** Percentages add up to >100% because resident-years could have been prescribed more than one antibiotic class. See eTable 3 for the list of antibiotics included in each class. | | |

**eTable 6. Association between prescriber type and 14-day outcomes among nursing home residents prescribed antibiotics for urinary tract infection, before and after IPTW, N= 106,354 resident-years.**

| **Outcome** | **Prescriber** **Type** | **Crude Risk Difference (95% CLs), Percentage Points** | **IPTW Risk Difference (95% CLs), Percentage Points** | **Crude Risk Ratio (95% CLs)** | **IPTW Risk Ratio (95% CLs)** |
| --- | --- | --- | --- | --- | --- |
| **Death** | Clinicians who did not primarily practice in NHs | Reference | Reference | Reference | Reference |
|  | Clinicians who primarily practiced in NHs | 0.05 (-0.01, 0.12) | 0.05 (-0.02, 0.12) | 1.28 (0.96, 1.70) | 1.26 (0.88, 1.64) |
|  |  |  |  |  |  |
| **All-Cause Hospitalization** | Clinicians who did not primarily practice in NHs | Reference | Reference | Reference | Reference |
|  | Clinicians who primarily practiced in NHs | 0.52* (0.21, 0.82) | 0.10 (-0.22, 0.42) | 1.10* (1.04, 1.17) | 1.02 (0.96, 1.08) |
|  |  |  |  |  |  |
| **Hospitalization for UTI or Septicemia**^a^ | Clinicians who did not primarily practice in NHs | Reference | Reference | Reference | Reference |
|  | Clinicians who primarily practiced in NHs | 0.24* (0.03, 0.44) | 0.00 (-0.21, 0.22) | 1.11* (1.02, 1.21) | 1.00 (0.91, 1.10) |
|  |  |  |  |  |  |
| **Subsequent Antibiotic Dispensing** | Clinicians who did not primarily practice in NHs | Reference | Reference | Reference | Reference |
|  | Clinicians who primarily practiced in NHs | 0.75* (0.10, 1.41) | 0.80* (0.08, 1.51) | 1.02* (1.00, 1.04) | 1.02* (1.00, 1.04) |
| **Abbreviations**: CLs, confidence limits; IPTW, inverse probability of treatment weighting; NH, nursing home; UTI, urinary tract infection.  **Note**: Clinicians who primarily practiced in nursing homes were defined as prescribers with ≥90% of all dispensings (not only antibiotics) to nursing home residents. Clinician type was calculated yearly. We sampled one pneumonia/UTI infection per resident per year, so residents could be represented multiple times during the study period.  *p-value <0.05 | | | | | |

**eTable 7. Association between prescriber type and 14-day outcomes among nursing home residents prescribed antibiotics for pneumonia, before and after IPTW, N= 28,826 resident-years.**

| **Outcome** | **Prescriber** **Type** | **Crude Risk Difference (95% CLs), Percentage Points** | **IPTW Risk Difference (95% CLs), Percentage Points** | **Crude Risk Ratio (95% CLs)** | **IPTW Risk Ratio (95% CLs)** |
| --- | --- | --- | --- | --- | --- |
| **Death** | Clinicians who did not primarily practice in NHs | Reference | Reference | Reference | Reference |
|  | Clinicians who primarily practiced in NHs | 0.41* (0.12, 0.70) | 0.36* (0.07, 0.65) | 1.36* (1.11, 1.68) | 1.32* (1.04, 1.60) |
|  |  |  |  |  |  |
| **All-Cause Hospitalization** | Clinicians who did not primarily practice in NHs | Reference | Reference | Reference | Reference |
|  | Clinicians who primarily practiced in NHs | -1.32* (-1.97,  -0.66) | -1.30* (-1.97,  -0.63) | 0.85* (0.78, 0.92) | 0.85* (0.77, 0.92) |
|  |  |  |  |  |  |
| **Hospitalization for Pneumonia or Septicemia** | Clinicians who did not primarily practice in NHs | Reference | Reference | Reference | Reference |
|  | Clinicians who primarily practiced in NHs | -0.64* (-1.11,  -0.17) | -0.71* (-1.19,  -0.24) | 0.85* (0.75, 0.96) | 0.83* (0.72, 0.94) |
|  |  |  |  |  |  |
| **Subsequent Antibiotic Dispensing** | Clinicians who did not primarily practice in NHs | Reference | Reference | Reference | Reference |
|  | Clinicians who primarily practiced in NHs | 0.22 (-0.96, 1.40) | 0.77 (-0.43, 1.98) | 1.01 (0.97, 1.04) | 1.02 (0.99, 1.05) |
| **Abbreviations**: CLs, confidence limits; IPTW, inverse probability of treatment weighting; NH, nursing home.  **Note**: Clinicians who primarily practiced in nursing homes were defined as prescribers with ≥90% of all dispensings (not only antibiotics) to nursing home residents. Clinician type was calculated yearly. We sampled one pneumonia/UTI infection per resident per year, so residents could be represented multiple times during the study period.  *p-value <0.05 | | | | | |

**eTable 8. E-values for 14-day outcomes.**

| **Outcome** | **Crude** | | **IPTW** | |
| --- | --- | --- | --- | --- |
|  | **Risk Ratio (95% CLs)** | **E-value, Point Estimate (CL)^a^** | **Risk Ratio (95% CLs)** | **E-value, Point Estimate (CL)^a^** |
| **Note: Reference group is clinicians who did not primarily practice in nursing homes** | | | | |
| **Death** | | | | |
| UTI | 1.28 (0.96, 1.70) | 1.88 (1.00**) | 1.26 (0.88, 1.64) | 1.83 (1.00**) |
| Pneumonia | 1.36 (1.11, 1.68) | 2.06 (1.46) | 1.32 (1.04, 1.60) | 1.97 (1.24) |
| **All-Cause Hospitalization** | | | | |
| UTI | 1.10 (1.04, 1.17) | 1.43 (1.24) | 1.02 (0.96, 1.08) | 1.16 (1.00**) |
| Pneumonia | 0.85 (0.78, 0.92) | 1.63 (1.39) | 0.85 (0.77, 0.92) | 1.63 (1.39) |
| **Hospitalization for Infection or Septicemia** | | | | |
| UTI | 1.11 (1.02, 1.21) | 1.46 (1.16) | 1.00 (0.91, 1.10) | 1.00 (1.00**) |
| Pneumonia | 0.85 (0.75, 0.96) | 1.63 (1.25) | 0.83 (0.72, 0.94) | 1.70 (1.32) |
| **Subsequent Antibiotic Dispensing** | | | | |
| UTI | 1.02 (1.00, 1.04) | 1.16 (1.00**) | 1.02 (1.00, 1.04) | 1.16 (1.00**) |
| Pneumonia | 1.01 (0.97, 1.04) | 1.11 (1.00**) | 1.02 (0.99, 1.05) | 1.16 (1.00**) |
| **Abbreviations**: CL, confidence limit; IPTW, inverse probability of treatment weighted; UTI, urinary tract infection.  ^a^It is only meaningful to calculate the E-value for the confidence limit closest to the null (RR=1).  **Value is 1 because RR 95% CL estimate includes 1. | | | | |

**eTable 9. Unadjusted 30-day outcomes for nursing home residents prescribed antibiotics for urinary tract infection or pneumonia, by prescriber type, 2016-2018.**

|  | **n (%)** | | | |
| --- | --- | --- | --- | --- |
|  | **UTI** | | **Pneumonia** | |
| **Outcome** | **Clinicians who did not primarily practice in NHs (n=77,799 resident-years)** | **Clinicians who primarily practiced in NHs (n=28,555 resident-years)** | **Clinicians who did not primarily practice in NHs (n=19,282 resident-years)** | **Clinicians who primarily practiced in NHs (n=9,544 resident-years)** |
| **Death** | 815 (1.05%) | 320 (1.12%) | 805 (4.17%) | 439 (4.60%) |
| **All-Cause Hospitalization** | 6,905 (8.88%) | 2,782 (9.74%) | 2,538 (13.16%) | 1,111 (11.64%) |
| **Hospitalization for Infection or Septicemia^a^** | 3,017 (3.88%) | 1,239 (4.34%) | 1,177 (6.10%) | 514 (5.39%) |
| **Subsequent Antibiotic Dispensing** | 40,930 (52.61%) | 14,767 (51.71%) | 8,881 (46.06%) | 4,350 (45.58%) |
| **Abbreviations**: NH, nursing home; UTI, urinary tract infection.  **Note**: Clinicians who primarily practiced in nursing homes were defined as prescribers with ≥90% of all dispensings (not only antibiotics) to nursing home residents. Clinician type was calculated yearly. We sampled one pneumonia/UTI infection per resident per year, so residents could be represented multiple times during the study period.  ^a^Outcome for the UTI group included hospitalization for UTI or septicemia; outcome for the pneumonia group included hospitalization for pneumonia or septicemia. | | | | |

**eTable 10. Association between prescriber type and 30-day outcomes among nursing home residents prescribed antibiotics for urinary tract infection, before and after IPTW, N= 106,354 resident-years.**

| **Outcome** | **Prescriber** **Type** | **Crude Risk Difference (95% CLs), Percentage Points** | **IPTW Risk Difference (95% CLs), Percentage Points** | **Crude Risk Ratio (95% CLs)** | **IPTW Risk Ratio (95% CLs)** |
| --- | --- | --- | --- | --- | --- |
| **Death** | Clinicians who did not primarily practice in NHs | Reference | Reference | Reference | Reference |
|  | Clinicians who primarily practiced in NHs | 0.07 (-0.07, 0.21) | 0.08 (-0.08, 0.23) | 1.07 (0.94, 1.22) | 1.07 (0.92, 1.22) |
|  |  |  |  |  |  |
| **All-Cause Hospitalization** | Clinicians who did not primarily practice in NHs | Reference | Reference | Reference | Reference |
|  | Clinicians who primarily practiced in NHs | 0.87* (0.47, 1.26) | 0.20 (-0.22, 0.62) | 1.10* (1.05, 1.14) | 1.02 (0.98, 1.07) |
|  |  |  |  |  |  |
| **Hospitalization for UTI or Septicemia** | Clinicians who did not primarily practice in NHs | Reference | Reference | Reference | Reference |
|  | Clinicians who primarily practiced in NHs | 0.46* (0.19, 0.73) | 0.11 (-0.18, 0.40) | 1.12* (1.05, 1.19) | 1.03 (0.96, 1.10) |
|  |  |  |  |  |  |
| **Subsequent Antibiotic Dispensing** | Clinicians who did not primarily practice in NHs | Reference | Reference | Reference | Reference |
|  | Clinicians who primarily practiced in NHs | -0.90* (-1.57,  -0.22) | -0.21 (-0.94, 0.52) | 0.98* (0.97, 1.00) | 1.00 (0.98, 1.01) |
| **Abbreviations**: CLs, confidence limits; IPTW, inverse probability of treatment weighting; NH, nursing home; UTI, urinary tract infection.  **Note**: Clinicians who primarily practiced in nursing homes were defined as prescribers with ≥90% of all dispensings (not only antibiotics) to nursing home residents. Clinician type was calculated yearly. We sampled one pneumonia/UTI infection per resident per year, so residents could be represented multiple times during the study period.  *p-value <0.05 | | | | | |

**eTable 11. Association between prescriber type and 30-day outcomes among nursing home residents prescribed antibiotics for pneumonia, before and after IPTW, N= 28,826 resident-years.**

| **Outcome** | **Prescriber** **Type** | **Crude Risk Difference (95% CLs), Percentage Points** | **IPTW Risk Difference (95% CLs), Percentage Points** | **Crude Risk Ratio (95% CLs)** | **IPTW Risk Ratio (95% CLs)** |
| --- | --- | --- | --- | --- | --- |
| **Death** | Clinicians who did not primarily practice in NHs | Reference | Reference | Reference | Reference |
|  | Clinicians who primarily practiced in NHs | 0.42 (-0.08, 0.93) | 0.45 (-0.06, 0.97) | 1.10 (0.98, 1.23) | 1.11 (0.98, 1.24) |
|  |  |  |  |  |  |
| **All-Cause Hospitalization** | Clinicians who did not primarily practice in NHs | Reference | Reference | Reference | Reference |
|  | Clinicians who primarily practiced in NHs | -1.52* (-2.32,  -0.72) | -1.55* (-2.37, -0.73) | 0.88* (0.83, 0.94) | 0.88* (0.82, 0.94) |
|  |  |  |  |  |  |
| **Hospitalization for Pneumonia or Septicemia** | Clinicians who did not primarily practice in NHs | Reference | Reference | Reference | Reference |
|  | Clinicians who primarily practiced in NHs | -0.72* (-1.28,  -0.15) | -0.87* (-1.45, -0.29) | 0.88* (0.80, 0.98) | 0.86* (0.77, 0.95) |
|  |  |  |  |  |  |
| **Subsequent Antibiotic Dispensing** | Clinicians who did not primarily practice in NHs | Reference | Reference | Reference | Reference |
|  | Clinicians who primarily practiced in NHs | -0.48 (-1.70, 0.74) | 0.33 (-0.92, 1.58) | 0.99 (0.96, 1.02) | 1.01 (0.98, 1.03) |
| **Abbreviations**: CLs, confidence limits; IPTW, inverse probability of treatment weighting; NH, nursing home.  **Note**: Clinicians who primarily practiced in nursing homes were defined as prescribers with ≥90% of all dispensings (not only antibiotics) to nursing home residents. Clinician type was calculated yearly. We sampled one pneumonia/UTI infection per resident per year, so residents could be represented multiple times during the study period.  *p-value <0.05 | | | | | |

**eTable 12. Association between prescriber type and 14-day outcomes among nursing home residents prescribed antibiotics for pneumonia, after IPTW and IPCW due to death, N= 28,826 resident-years.**

| **Outcome** | **Prescriber** **Type** | **Censored due to death, n (%)** | **Adjusted Risk Difference (95% CLs), Percentage Points** | **Adjusted Risk Ratio**  **(95% CLs)** |
| --- | --- | --- | --- | --- |
| **All-Cause Hospitalization** | Clinicians who did not primarily practice in NHs | 166 (0.86) | Reference | Reference |
|  | Clinicians who primarily practiced in NHs | 113 (1.18) | -1.28* (-1.96, -0.60) | 0.85* (0.78, 0.93) |
|  |  |  |  |  |
| **Hospitalization for Pneumonia or Septicemia** | Clinicians who did not primarily practice in NHs | 190 (0.99) | Reference | Reference |
|  | Clinicians who primarily practiced in NHs | 128 (1.34) | -0.72* (-1.20, -0.23) | 0.83* (0.73, 0.95) |
|  |  |  |  |  |
| **Subsequent Antibiotic Dispensing** | Clinicians who did not primarily practice in NHs | 134 (0.69) | Reference | Reference |
|  | Clinicians who primarily practiced in NHs | 82 (0.86) | 0.91 (-0.34, 2.16) | 1.03 (0.99, 1.06) |
| **Abbreviations**: CLs, confidence limits; IPTW, inverse probability of treatment weighting; IPCW, inverse probability of censoring weighting; NH, nursing home.  **Note**: Clinicians who primarily practiced in nursing homes were defined as prescribers with ≥90% of all dispensings (not only antibiotics) to nursing home residents. Clinician type was calculated yearly. We sampled one pneumonia/UTI infection per resident per year, so residents could be represented multiple times during the study period.  *p-value <0.05 | | | | |
